# The case against stenting the cannulation zone of dialysis access

**DOI:** 10.1016/j.radcr.2024.08.141

**Published:** 2024-09-16

**Authors:** Ivana Boktor, Abduljalil Alragheb, Ahmed E. Ali, Ammar Almehmi

**Affiliations:** a George Walton Comprehensive High School, Marietta, GA, USA; b University of Sharjah, University City, Sharjah, UAE; c Internal Medicine Residency Program, Crestwood Medical Center, Huntsville, AL, USA; d Department of Medicine and Radiology, University of Alabama at Birmingham, Birmingham, AL, USA

**Keywords:** Vascular access, Dialysis, Stent graft, Arteriovenous graft

## Abstract

The indications for stent grafts (SG) placement within the dialysis vascular access include recurrent stenosis at the venous anastomosis of arteriovenous grafts, vessel rupture, pseudoaneurysm exclusion, and intra-stent stenosis. Controversy exists regarding the use of SGs within the cannulation zone due to the theoretical risks of increased infection and stent fracture. While prospective studies are lacking, several retrospective studies demonstrated the safety of SG use within the cannulation area. However, the short-term nature of these retrospective studies makes it challenging to draw any reasonable conclusions about SG's long-term safety profile. The presented case here showed that the accumulative exposure to needle injury during dialysis therapy was associated with fracturing the stent leading to life-threatening skin ulcerations that required immediate surgical intervention. Additionally, this case suggests that deploying SG within the cannulation segment should be reserved for those with poor survival and have exhausted other access options.

## Introduction

Stent grafts (SG) are commonly used to manage vascular access dysfunction including recurrent stenosis, thrombosis, and occasionally, access aneurysms. The premise of using SGs is to prolong the dialysis access life span among end-stage renal disease (ESRD) patients who are on dialysis [1,2]. However, the off-label use of SGs within the cannulation zone remains controversial. This controversy stems from the fact that repeated puncture of SG using the large bore dialysis needles can damage the stent structure [1], which could potentially increase the risk of stent fractures, kinks, skin erosion, and subsequent ulceration [3]. Despite these risks, however, several retrospective studies reported the feasibility and effectiveness of using SGs within the cannulation zone to salvage the dialysis access without any major complications [4].

Here we present an unusual case of left upper arm arteriovenous graft (AVG) that had multiple stents in a dialysis patient who presented with skin ulceration and impending rupture at the site of old fractured stents within the dialysis access, which required urgent surgery.

## Case description

A 64-year-old male with a history of hypertension, atrial fibrillation on chronic oral anticoagulation, diastolic heart failure, mitral valve replacement, and ESRD secondary to biopsy-proven focal segmental glomerulosclerosis, presented to our center with 2 ulcerated areas over his left upper arm AVG.

**Fig. 1:**
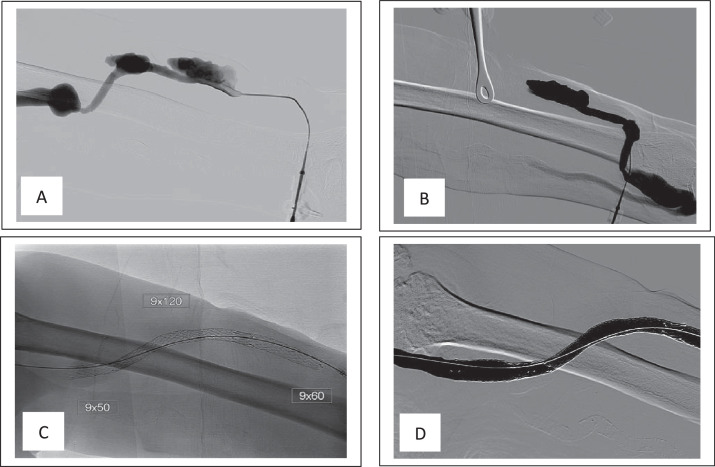
(A) graft angiogram showing 2 aneurysmal formations. (B) angiogram showing the arterial anastmosis and connection between cephalic vein and interpositioning graft. (C) single shot X-ray showing 3 overlapping stents. (D) poststenting angiogram revealing the exclusion of the aneurysmal formations.

**Fig. 2:**
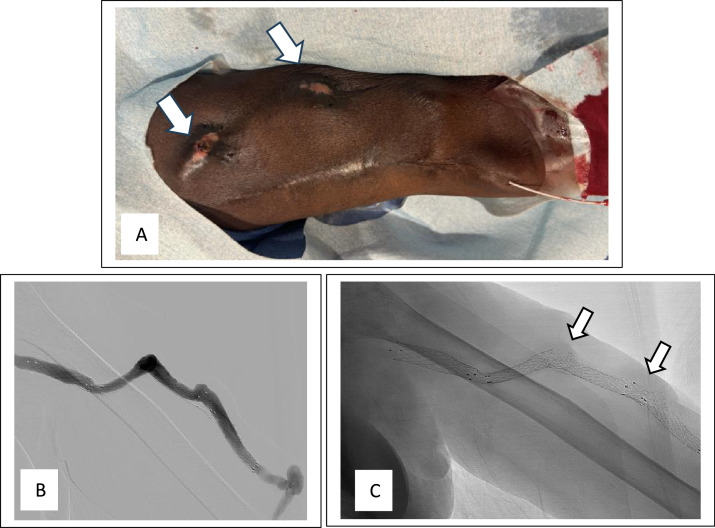
(A) skin ulcerated areas corresponding with the cannulation areas/fractured stents (arrows). (B) graft angiogram. (C) single shot X-ray showing fractured stents (arrows).

In the background, the patient started dialysis via a left upper arm brachiocephalic AV fistula created in January 2010. Over time, multiple pseudoaneurysms had developed within the AV fistula body that were repaired with surgical resection and placement of an interpositioning AVG in April 2012. However, in September 2017, the interpositioning AVG developed multiple pseudoaneurysmal formations within the graft body that were treated with several covered stents to exclude these formations (Fig. 1). The patient lost follow-up until June 2024 when he presented to our facility with skin ulcers and high venous pressure during dialysis. Physical examination revealed 2 areas of skin discoloration with an eschar covering the upper lesion (Fig. 2A). The AVG angiogram revealed multiple stents within the dialysis access with 2 stent fractures noted at the cannulation sites, corresponding to repetitive needle cannulation and skin lesions (Figs. 2B and C). The overlapping skin of the ulcerative lesions was very thin on palpation. The patient was taken for emergent access excision due to the increased risk of access impending rupture. Concurrently, a new AV fistula was created in the right upper extremity and a central venous catheter was inserted to resume hemodialysis in the meantime.

## Discussion

Over the last decade, the use of SG in managing dialysis access lesions has expanded with the goal to extend the survival of these accesses. Most of the clinical trials were designed to assess the effect of SG on target lesion patency rate and overall dialysis access patency rate [2]. Historically, the use of SGs in the cannulation zone was avoided due to the associated risks and complications such as stent fracture, infection, and thrombosis. However, accumulative evidence showed that SGs can be used safely within the cannulation segment without adverse effects. Hence, if required, the use of SGs can be justified especially when the dialysis access loss is at risk. For instance, Aronhime et al. [4] reviewed 40 patients (14 AVGs and 26 AVFs) who underwent SG placement within the cannulation zone and showed a primary and secondary patency rate of the target lesion at 12 months of 74% and 78%, respectively. In another study, Rhodes et al. [5] assessed 11 patients with AVGs who were treated with SGs to exclude AVG aneurysms and reported a secondary patency rate at 9 months of 100% with no recorded complications. Moreover, Drouven et al. [6] reviewed 11 patients with AVGs who were treated with SGs due to recurrent stenotic lesions within the cannulation zone and reported a secondary patency rate of 80% at 24 months.

As stated earlier, the main concerns about deploying SGs within the cannulation zone include increased theoretical risk of access infection, thrombosis, and stent fracture. While these complications were not encountered in the previous studies, Wong et al. [7] followed 35 patients who underwent SGs placement in the cannulation segment to manage AVG pseudoaneurysm and reported a 17.1% infection rate and 37.1% thrombosis rate.

It is worth mentioning that these studies did not show any increase in the stent fracture risk likely due to their short follow-up, limited mostly to 2 years. Thus, the long-term complications of SGs, namely fractures, are yet to be elucidated. It is plausible to postulate that the longer the exposure to repeated trauma during needle cannulation, the higher the risk for stent fracture. This possibility was demonstrated in our case who had an accumulated time of cannulation of more than 5 years resulting in the loss of stent integrity and subsequent strut protrusion through the overlapping tissue leading to skin ulceration.

In conclusion, this case highlights the need for careful consideration of the risks and benefits of SG placement in managing dialysis access complications (stenosis, aneurysms, and thrombosis). Perhaps, SG deployment within the cannulation zone may be reserved for elderly dialysis patients who exhausted access options and have limited life expectancy. Otherwise, surgical revision or creating a new access may be the preferred approach to achieve better long-term outcomes and avoid potentially serious complications [8].

## Patient consent

Written informed consent was obtained from the patient for all procedures and publication of this case and accompanying images.
